# Direct Medial Entorhinal Cortex Input to Hippocampal CA3 Is Crucial for eEF2K Inhibitor-Induced Neuronal Oscillations in the Mouse Hippocampus

**DOI:** 10.3389/fncel.2020.00024

**Published:** 2020-03-06

**Authors:** Ziyang Liu, Cheng Peng, Yinghan Zhuang, Ying Chen, Thomas Behnisch

**Affiliations:** Institutes of Brain Science, State Key Laboratory of Medical Neurobiology and MOE Frontiers Center for Brain Science, Fudan University, Shanghai, China

**Keywords:** hippocampus, calcium imaging, oscillation, MEC, eEF2K inhibitor

## Abstract

The hippocampal formation plays a vital role in memory formation and takes part in the control of the default neuronal network activity of the brain. It also represents an important structure to analyze drug-induced effects on subregion-specific synchronization of neuronal activity. However, the consequences of an altered functional state of synapses for subregion-specific synchronization of neuronal microcircuits remain to be fully understood. Therefore, we analyzed the direct interaction of neuronal microcircuits utilizing a genetically encoded calcium sensor (GCaMP6s) and local field potential (LFP) recording in acute hippocampal–entorhinal brain slices in response to a modulator of synaptic transmission. We observed that application of the eukaryotic elongation factor-2 kinase (eEF2K) inhibitor A484954, induced a large-scale synchronization of neuronal activity within different regions of the hippocampal formation. This effect was confirmed by the recording of extracellular LFPs. Further, in order to understand if the synchronized activity depended on interconnected hippocampal areas, we lesioned adjacent regions from each other. These experiments identified the origin of A484954-induced synchronized activity in the hippocampal CA3 subfield localized near the hilus of the dentate gyrus. Remarkably, the synchronization of neuronal activity in the hippocampus required an intact connection with the medial entorhinal cortex (MEC). In line with this observation, we detected an increase in neuronal activity in the MEC area after application of A484954. In summary, inhibition of eEF2K alters the intrinsic activity of interconnected neuronal microcircuits dominated by the MEC–CA3 afferents.

## Introduction

The hippocampal formation plays a vital role in memory formation and takes part in the control of the default cerebral network activity (Greicius et al., 2004; Basu and Siegelbaum, 2015; Vatansever et al., 2015; Schröter et al., 2017). This could mean that potential treatments for neurological disorders not only have a cellular component but could also affect brain-wide neuronal network oscillations (Greicius et al., 2004; Figueroa et al., 2017; Muthukumaraswamy and Liley, 2018; Moda-Sava et al., 2019). However, the consequences of an altered functional state of synapses for subregion-specific synchronization of neuronal microcircuits and interconnected brain areas are not completely understood.

Many studies have reported that certain frequencies of oscillations are particularly associated with some specific brain functions (Buzsáki and Draguhn, 2004; Osipova et al., 2006; Palva and Palva, 2007). In addition, brain oscillatory activity is not only a byproduct of neural activity but also has been linked with information encoding (Engel et al., 2001; Varela et al., 2001; Lisman and Jensen, 2013). Therefore, brain oscillatory activity performs an important role in regulating information flow between different brain regions (Buzsáki and Draguhn, 2004; Basu and Siegelbaum, 2015; Bush and Burgess, 2019). Such information flow is likely altered in patients with neuropsychiatric disorders, such as depression, because of their significantly different intrinsic oscillations between various brain areas (D’ostilio and Garraux, 2016; Li et al., 2017). In addition, many studies have shown that pharmacological antidepressant therapies or brain stimulation therapy rapidly relieves symptoms by altering brain-wide network activity (D’ostilio and Garraux, 2016; Dunlop et al., 2017; Kubicki et al., 2019). For example, the antidepressant drug ketamine and corresponding ketamine metabolites cause rapid antidepressant action and modulate reward-related neuronal plasticity (Yao et al., 2018) that tightly correlates with changes in brain-wide oscillation pattern (Bonhomme et al., 2016; Muthukumaraswamy and Liley, 2018). The cellular mechanisms of antidepressant therapies remain to be fully understood, but it has been suggested that one of the cellular mechanisms is linked to the modulation eEF2K activity (Zanos et al., 2016; Adaikkan et al., 2018; Sattar et al., 2018). The eukaryotic elongation factor-2 kinase (eEF2K), also known as calcium/calmodulin-dependent protein kinase III (CaMKIII), participates in the posttranscriptional regulation of mRNA translation (Liu and Proud, 2016). If the activity of eEF2K (Wang et al., 2001; Knebel et al., 2002) is attenuated, and then a reduced phosphorylation level of the elongation factor 2 (eEF2) promotes mRNA translation. Thus, the phosphorylation level of the eEF2 plays an important role in the regulation of the translational capacity within neurons (Heise et al., 2014). Some studies have found that eEF2K can effectively regulate the synaptic transmission of γ-aminobutyric acid (GABA), affect the excitation/inhibition balance of neurons, and therefore regulate cerebral oscillation (Heise et al., 2017). Another study described that specific inhibition of eEF2K in primary hippocampal cell cultures causes irregular neuronal activity to become synchronized (Weng et al., 2016). Moreover, the maintenance of hippocampal long-term plasticity depends under many conditions upon protein synthesis (Frey et al., 1988; Ris et al., 2009). In this respect, the involvement of eEF2K in neuronal plasticity has been shown to play a role in the regulation of dendritic protein synthesis in response to the history of synaptic activity (Kang and Schuman, 1996; Taha et al., 2013; McCamphill et al., 2015; Zimmermann et al., 2018).

The hippocampal formation is an important brain area involved in the regulation of brain-wide oscillation pattern and in the complex system of memory formation (Buzsáki and Moser, 2013; Basu and Siegelbaum, 2015). The hippocampal formation is, furthermore, an ideal brain area for studying the modulation of neuronal network oscillation (Dragoi et al., 1999) in response to an interference with synaptic transmission (Kohara et al., 2014; Butler and Paulsen, 2015). Hippocampal oscillation has been composed into several components: large amplitude irregular activity (0.5–20 Hz), rhythmic slow activity or theta (4–10 Hz), fast oscillatory activity (gamma, 30–100 Hz), and sharp wave/ripple complexes (Buzsáki and Draguhn, 2004; Maier and Kempter, 2017). In human, large-amplitude, irregular activity mainly occurs during quiescent awake states and slow-wave sleep, whereas theta and gamma rhythms mainly occur during REM sleep and exploratory activities (Uchida et al., 2001). In the past few decades, a number of studies have been conducted to study the cellular mechanism of these oscillations in the hippocampus by using hippocampal slices *in vitro* (Butler and Paulsen, 2015). Interestingly, in acute slices of the hippocampal formation, neuronal frequency patterns can be observed such as delta (0.5 to <4 Hz; Zhang et al., 1998), theta (4–10 Hz; Kang et al., 2015), and gamma (30–100 Hz; Bathellier et al., 2008; Butler and Paulsen, 2015).

In our previous work, we have shown that an eEF2K inhibitor induces the potentiation of hippocampal synaptic transmission and synchronizes the network activity of neurons in primary hippocampal cell cultures (Weng et al., 2016). However, it had not been studied whether such synchronization also takes place in interconnected hippocampal–entorhinal acute slices. In this study, we show that inhibition of eEF2K in hippocampal slices *in vitro* induces hippocampal neuronal network oscillation that is strongly dependent on the interconnected entorhinal cortex.

## Materials and Methods

### Animals

C57BL/6 mice (male, 6–10 weeks old, 20–25 g) were provided by the Department of Laboratory Animal Science of Fudan University, Shanghai, China. Animals were housed with a 12-h reverse dark-light cycle at 23°C and with free access to food and water. Efforts were made to minimize the number of animals sacrificed. This study was carried out in accordance with the recommendations of the Institutes of Brain Science and State Key Laboratory of Medical Neurobiology of Fudan University, Shanghai, China, and approved by the Institutional Animal Care and Use Committee of Fudan University, Shanghai Medical College (IACUC Animal Project no. 31320103906). The protocol was approved by the Institutes of Brain Science, Fudan University.

### Types of Adeno-Associated Viruses

The following viral titers were obtained from Shanghai Shengbo: AAV9-hSyn-GCaMP6s, AAV9-CaMKII-GCaMP6s. The titers were diluted to 5 × 10^12^ to 10 × 10^12^ VG/ml with iso-osmotic phosphate-buffered saline solution and injected within 1 week.

### Stereotaxic Injection of Adeno-Associated Virus

Mice were anesthetized by intraperitoneal administration of 2.5% tribromoethanol (Avertin, injected: 0.1 ml/10 g) and 3 mg/ml xylazine (injected: 0.04 ml/10 g). Aureomycin eye ointment was applied on both corneas of mice to prevent dry eyes from dehydration. For stereotaxic intracranial injection of viral vectors, the anesthetized animal was placed into a stereotaxic rack to allow precise insertion of glass pipettes into the intermediate hippocampus (Cetin et al., 2006). The coordinates of the different pipette positions were for the CA1: AP, 0.35; ML, 0.36; and DV, 0.30, and for the CA3: AP, 0.30; ML, 0.30; and DV, 0.30, and for the medial entorhinal cortex (MEC): AP, 0.40; ML, 0.40; and DV, 0.28 (cm). After reaching the final position with the pipette, the viral titer (5 × 10^12^ to10 × 10^12^ VG/ml) was injected at 0.5 μl per 5 min and additionally two times after sequential upward movements (100 μm each) of the pipette. The procedure was repeated at the other brain hemisphere. The animals were maintained on a heating board during the procedure and recovery from anesthetization.

### Hippocampal Slice Preparation

Acute hippocampal slices were prepared from 4- to 8-week-old male mice as described previously (Huang et al., 2015; Weng et al., 2016; Wang et al., 2017; Yun et al., 2018; Li et al., 2019) with slight modifications to ensure the connectivity of the hippocampal formation with the entorhinal cortex *in vitro* according to Xiong et al. (2017). Briefly, after anesthesia with isoflurane, the brains were isolated and immersed in pre-carbogenated (95% O_2_/5% CO_2_) ice-cold slicing salt buffer solution (composition in mM: 92 NaCl, 2.5 KCl, 0.5 CaCl_2_·2H_2_O, 10 MgSO_4_, 1.25 NaH_2_PO_4_, 25 glucose, 30 NaHCO_3_, 3 sodium pyruvate, 20 HEPES, titrated to pH 7.4; Pan et al., 2015). A piece of the dorsal cortex was sliced off and the two hemispheres glued with the created surface on the slicing platform of the sectioning system. Transverse hippocampal slices (350 μm) were cut (Vibratome; Leica Wetzlar, Germany) and maintained in a submerged-incubation chamber for at least 2 h at room temperature (25°C) before the transfer to a submerged-type recording chamber (RC-26GLP; Warner, Hamden, CT, USA) mounted on a Nikon (Nikon Instruments Inc., Melville, NY, USA) microscope stage (Weng et al., 2018). The slices rested for at least 30 min at 32°C under constant perfusion with pre-carbogenated artificial cerebrospinal fluid (aCSF; 4 ml/min). The aCSF used for the incubation and experiment consisted of (in mM) 124 NaCl, 2.5 KCl, 2 CaCl_2_·2H_2_O, 2 MgCl_2_·7H_2_O, 1.25 NaH_2_PO_4_, 10 D-glucose, 26 NaHCO_3_, pH 7.4).

### Field Potential Recordings

Spontaneous local field potentials (LFPs) were recorded using glass pipettes (2–3 MΩ) filled with aCSF. The potentials were 100 times amplified and filtered by an Axon amplifier (MultiClamp 700B; Molecular Devices, San Jose, CA, USA; 0.1-Hz high-pass filter; 3-kHz low-pass filter) and then digitized at a sample frequency of 20 kHz by an AD/DA converter software system (Digidata 1400, Molecular Devices, San Jose, CA, USA).

### Fluorescence Imaging

The fluorescence of the GCaMP6s was evoked at 488 nm generated by a TTL-controlled LED light source (X-Cite 120LED; Excelitas, Waltham, Massachusetts, USA) mounted on the EPI-fluorescence port of a microscope (Nikon Eclipse FN1 microscope; Nikon). The emission was acquired through a 16x water dipping objective (NA: 0.8, Nikon, Japan) by a PCO.Edge 5.5 sCMOS camera (PCO AG, Kelheim, Germany). The acquisition frame rate was 33 frames per seconds (FPS), and 3,000 frames were typically acquired.

### Analysis of Time-Lapse Fluorescence Images

**Figure 1:**
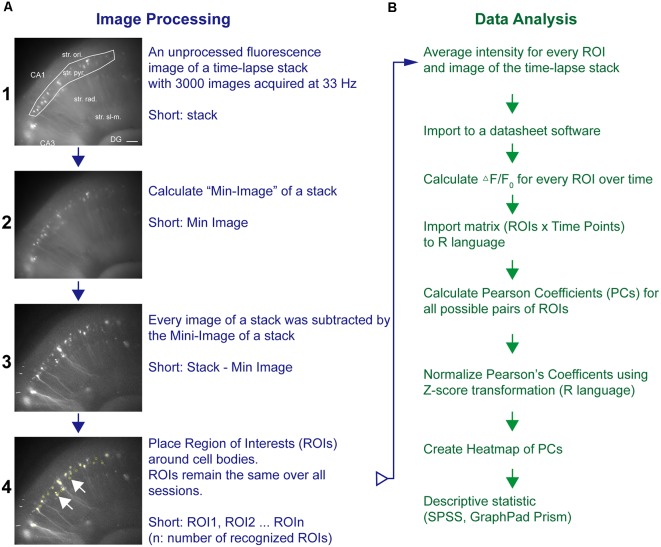
Pipeline of neuronal network analysis in hippocampal–entorhinal slices. The network analysis pipeline consists of **(A)** image processing and fluorescence intensity extraction followed by **(B)** cross-correlation analysis of fluorescence transients from different areas of a specific region. Fluorescence images in **(A)** represent representative examples after the respective imaging processing. The regions of interest (ROIs) were placed around neurons as shown in A4. Horizontal scale bar corresponds to 100 μm.

Time-lapse imaging data (stack, 640 × 540 pixel × 3,000 frames) were processed and analyzed by ImageJ (National Institutes of Health, Bethesda, MD, USA) as outlined in Figure 1. Briefly, a Min-Intensity Image (ImageJ: Z-Project-Min) over all images was subtracted from the unprocessed frames of the stack to adjust for background intensities (Piatkevich et al., 2019). Using ImageJ’s Time Series Analyzer V3 plugin, regions of interest (ROIs) were placed on randomly chosen fluorescent cell bodies along stratum pyramidale (str. pyr.) or granular cell layer (GCL) and the average pixel intensity (F) per time point for every ROI acquired (Figure 1A). To this end, the Stack-Min image has been used, and if the expression pattern allowed, more than 20 ROIs per experiment were chosen. The ROI coordinates were stored and reloaded for the analysis of the next sessions and thus kept constant over all sessions. The values of the first 2 s of a time-lapse sequence were averaged and taken as the baseline value *F*_0_. The background corrected fluorescence intensity changes are presented as (*F* − *F*_0_)/*F*_0_. Statistical software (SPSS, GraphPad Prism software; GraphPad Software Inc., La Jolla, CA, USA) was used to calculate Pearson correlation coefficients (PCs) for all ROI pairs. The PC has been applied as the main read-out parameter of these imaging experiments. The PC is widely used to address the degree of synchronization of time-series pairs. It is widely used in functional magnetic resonance imaging and calcium imaging studies to estimate the degree of synchronization and coupling of two different brain areas, ROIs, or neurons (Bastos and Schoffelen, 2015; Vatansever et al., 2015; Pachitariu et al., 2018). To compare the degree of synchronization, the PC has been calculated for every possible pair of ROIs. The single values were further averaged to yield the averaged PC value for a certain imaging session. The PCs were transformed by a Fisher *Z* transformation and presented in heat maps (ROIn × ROIn: color-coded *Z* score-transformed PC values; R-language; R-Foundation, Vienna, Austria) and further summarized to allow the comparison of different conditions (Figure 1B).

**Figure 2:**
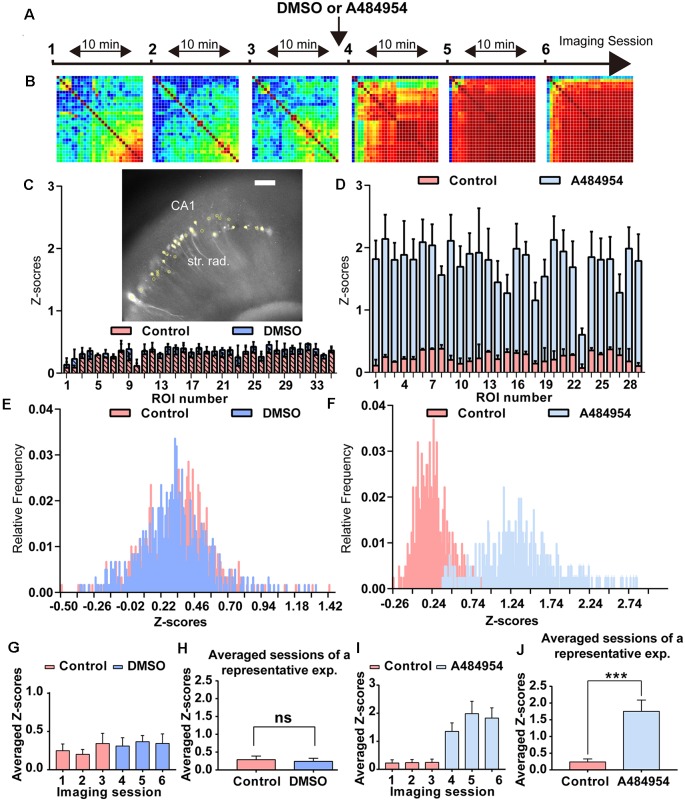
Analysis of the degree of cross-correlation between different areas of the CA1 region of representative time-lapse imaging experiments under control condition and after application of A484954. **(A)** The schema indicates the sequence of time-lapse fluorescence imaging and the time point of A484954 (10 μM) application. **(B)** Heat maps are presented for every imaging session showing color-coded the PC value for each ROI pair. Red corresponds to a value of 1 (maximal positive cross-correlation) and blue to a value of −1 (maximal negative cross-correlation). Under drug-free conditions, the imaging sessions revealed only a moderate cross-correlation between fluorescence transients of ROI pairs. However, after application of A484954, the number of correlative coupled pairs increased strongly. **(C)** The insert shows a representative hippocampal slice with its GCaMP6s-expressing CA1 neurons (white spots). The yellow filled circles indicate ROIs. The bar graph represents superimposed *Z* scores of PCs for each ROI under control (first three sessions, light pink) and dimethyl sulfoxide (DMSO) application (last three sessions, blue). **(D)** In a different representative example, the application of A484954 (10 μM) evoked an increase of cross-correlation coefficients in comparison with the drug-free sessions. The Z scores have been depicted in the bar graph for the two conditions. **(E)** The frequency distribution of *Z* scores has been presented in a histogram for sessions 3 and 6. **(F)** This histogram shows that the relative frequency distribution changed after drug application to the right. **(G)** For statistical comparison, the mean and SEM of *Z* scores over every pair were calculated and depicted in the bar graph for every session. **(H)** The bar graph shows that there is no difference in the average of *Z* scores of the first three control sessions (0.29 ± 0.10) followed by three DMSO sessions (0.24 ± 0.09; ns, paired *t*-test). **(I)** The bar graph summarizes the results for every session in the control–drug experiment. **(J)** The bar graph shows that averaged *Z* scores of three sessions differ significantly between the two conditions (control: 0.24 ± 0.09; A484954: 1.75 ± 0.34; ***P < 0.01, paired *t*-test).

To compare the previous approach with a different imaging analysis pipeline, the data in Figure 2 were analyzed using Suite2P (Pachitariu et al., 2018; Python package, Python Software Foundation, Wilmington, DE, USA) followed by a Granger-causality analysis (Luo et al., 2017). The analysis revealed similar changes of synchronized fluorescence changes over different ROIs in response to drug application (Pachitariu et al., 2018) and a CA3-Granger causality toward CA1 (Supplementary Figure S1).

### Compounds

Bicuculline and CNQX (Selleck, Shanghai, China) were dissolved freshly in aCSF. A484854 was dissolved in dimethyl sulfoxide (DMSO). The final concentration of DMSO (Sigma-Aldrich, Shanghai, China) was not higher than 0.05%, a concentration that has no effect on basal synaptic transmission and activity-dependent synaptic plasticity (Navakkode et al., 2004; Cai et al., 2010).

### Statistical Analysis

Paired and unpaired *t*-tests were applied for statistical comparison between the groups using SPSS [International Business Machines Corporation (IBM), Armonk, New York, USA] or GraphPad Prism software (GraphPad Software Inc.). *P < 0.05, **P < 0.01, and ***P < 0.001 indicate a statistically significant difference (ns: no significance). Control experiments mirrored the application time of compounds with 0.05% DMSO in aCSF.

## Results

### eEF2K Inhibition by A484954 Induces Synchronized Fluorescence Transients in the Hippocampus and Medial Entorhinal Cortex

As introduced before, the eEF2K inhibitor A484954 increases synaptic transmission in acute hippocampal slices and induces neuronal network synchronization in primary hippocampal cultures (Weng et al., 2016). To further detail the A484954 effect on neuronal network activity in a more complex environment, experiments were conducted in hippocampal–entorhinal acute slices. To this end, GCaMP6s-overexpressing acute slices were transferred to a slice chamber mounted on a video imaging fluorescence microscope. To learn about the intrinsic oscillations within the CA1 area of the hippocampus under control conditions and to compare with the effects of the solvent DMSO, the fluorescence changes were recorded repeatedly over several sessions (Figure 2A). A random activity of neurons was observable under baseline conditions. The neuronal activity was similar under control and DMSO administration conditions (Figure 2C), which is also documented in the *Z* score distribution histogram that indicates a similar distribution under control and DMSO (Figure 2E). The averaged *Z* scores for each session did not differ (Figure 2G). To simplify data analyses, all *Z* scores over all ROIs and sessions were averaged and presented in the bar diagram. There was no significant difference between the predosing (0.29 ± 0.10) and postdosing (0.24 ± 0.09; ns, paired *t*-test, Figure 2H). The values indicated that DMSO administration does not significantly alter the intrinsic activity of CA1 neurons.

To study the effects of the eEF2K inhibitor A484954 on area-dependent synchronicity in the hippocampal–entorhinal slices, similar recordings and analyses were conducted as described before (Figure 2A), with the exception of A484954 administration within the last three sessions. The application of A484954 induced a synchronization of fluorescence signals within different areas along the CA1 region.

A484954 enhanced the level of synchronized activities between ROI pairs as indicated in the heat maps (sessions 4–6) in Figure 2B. Moreover, the *Z* score frequency distribution showed a right shift, indicating that the level of synchronized activity increased (Figures 2D,F). The averaged *Z* scores over all ROI pairs further indicated the effect of A484954 on synchronization of fluorescence signals (Figure 2I). The *Z* score averages over all drug-free (0.24 ± 0.09) and drug sessions (1.75 ± 0.34) differed significantly (*P* < 0.01, paired *t*-test, Figure 2J).

The data set for the A484954 experiment was reanalyzed utilizing Suite2P, an imaging analysis package (Pachitariu et al., 2018). To be able to compare the activity within same ROIs at different sessions, the positions of the ROIs were chosen manually according to the correlation map. In addition, background subtracted fluorescence transients were deconvolved to obtain an estimation of underlying neuronal activity (Supplementary Figures S1A,B; Pachitariu et al., 2018). The fluorescence and deconvolved transients were nonsynchronized within different areas before the drug application (session 3, control); however, the signals synchronized in response to A485954 (session 6, Supplementary Figure S1B). The increase in synchronized fluorescence changes or estimated neuronal activity (deconvolved fluorescence transients) between pairs of ROIs is evident in the heat maps summarizing the PC values for different sessions (Supplementary Figure S1C). To estimate the causal interaction between ROI pairs, a Granger analysis of deconvolved transients has been performed (Luo et al., 2017). The calculation indicated that only areas at the border of the CA1 region interacted with the remaining ROIs (Supplementary Figure S1D). Overall Suite2P analysis confirmed the effects of A484954 on region synchronization of fluorescence transients.

**Figure 3:**
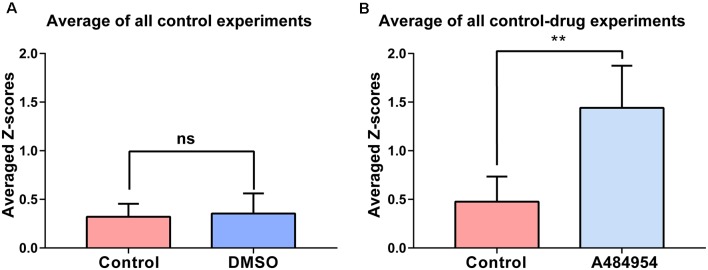
Synchronization level between different CA1 ROIs in response to DMSO or A484954. **(A)** The bar graph shows that DMSO (0.35 ± 0.07) did not alter the synchronization level between different CA1 areas in comparison with the control group (0.32 ± 0.05; *n* = 8, ns, unpaired *t*-test). **(B)** The bar graph shows that in all slices treated with A484954, the *Z* score—transformed PC values of the A484954 group (1.44 ± 0.15) were significantly increased compared with the control group (0.48 ± 0.09; *n* = 8, **P < 0.01, unpaired *t*-test).

The experiments that have been outlined above were repeated eight times. The basal neuronal activity and the degree of correlation between neuronal pairs were not affected by the application of DMSO (*n* = 8, ns, unpaired *t*-test between control (0.32 ± 0.05) and DMSO (0.35 ± 0.07, Figure 3A). In addition, these control experiments indicate the stability of synchronicity of different CA1 areas over a period of about 1 h. The averaged *Z* score-transformed PCs before (0.48 ± 0.09) and after A484954 (1.44 ± 0.15) differed significantly (*P* < 0.01, unpaired *t*-test, Figure 3B).

**Figure 4:**
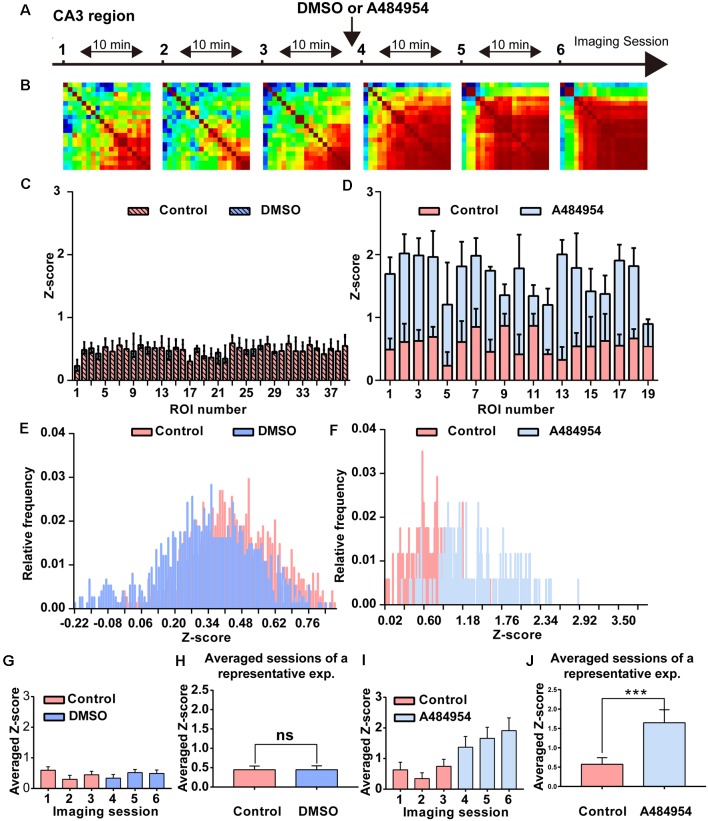
Detailed analysis of a time-lapse imaging experiment in the CA3 region under control–DMSO or control–A484954 conditions. **(A)** The schema indicates the sequence or time-lapse imaging session. The first three sessions were under control conditions, and the last three sessions under DMSO or A484954. **(B)** Heat maps (color-coded PC values per pair of ROIs) have been presented for the six sessions. The increase in red color indicates an enhancement of synchronized fluorescence transients after A484954 application. **(C)** The bar graph summarizes the individual *Z* scores for over 30 neuronal pairs for the two indicated conditions. The data indicated that DMSO did not affect the level of synchronization. **(D)** The level of synchronicity increased substantially after application of A484954 in comparison with the control sessions. **(E)** The frequency histograms for the *Z* scores of a control and DMSO session are presented. Only a slight shift to the left was observable after DMSO application. **(F)** After application of A484954, the *Z* score distribution shifted to the right, indicating a higher level of synchronicity within different CA3 areas. **(G)** The bar graph shows the averaged *Z* score over all ROI pairs for every session (session 1, 0.60 ± 0.11; session 2, 0.30 ± 0.13; session 3, 0.45 ± 0.11; session 4, 0.34 ± 0.12; session 5, 0.52 ± 0.10; session 6, 0.49 ± 0.12). **(H)** The values for the control–drug experiment are presented. After application of A484954, a tendency of increased *Z* scores was notable (session 1, 0.63 ± 0.25; session 2, 0.34 ± 0.19; session 3, 0.75 ± 0.22; session 4, 1.37 ± 0.35; session 5, 1.66 ± 0.36; session 6, 1.91 ± 0.42). **(I)** The bar graph shows the *Z* score-transformed PC values for each session under control and DMSO conditions (control: 0.45 ± 0.10; DMSO: 0.44 ± 0.11, ns, paired *t*-test). **(J)** The bar graph shows the values for three averaged sessions under control–A484954 conditions (control: 0.5 ± 1.64; A484954: 1.64 ± 0.33; ***P < 0.01, paired *t*-test).

Because inhibition of eEF2K induced a synchronization of neuronal activity in the hippocampal CA1 region, we wanted to study whether a similar synchronization of neuronal activity occurs in the CA3 region. To this end, the AAV9-hSyn-GCaMP6s was injected into the hippocampal CA3 region, and time-lapse fluorescence imaging experiments were performed 2 weeks later. We observed that 10 μM A484954 induced a large-scale synchronization of neuronal activity within the CA3 region as representatively indicated in the Supplementary Figures S2A–D. In addition, Figure 4 shows that fluorescence transients within several ROIs increased in a synchronized manner in response to the drug application. In contrast, application of the solvent DMSO did not alter the *Z* score distribution (Figures 4C,E,G,H). Further analysis of the representative experiment indicated that A484854 evoked synchronized fluorescence transients of a larger number of ROI pairs in comparison to the drug-free sessions (Pearson coefficient heat map, Figure 4A; *Z* score frequency distribution histogram Figure 4D) and average of all ROIs per session (0.57 ± 1.64, 1.64 ± 0.33, *P* < 0.01, paired *t*-test, Figure 4J).

**Figure 5:**
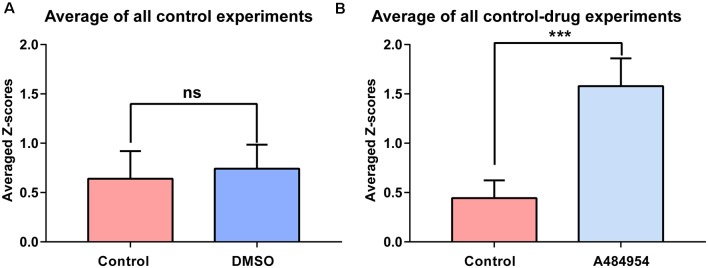
The effects of DMSO and A484954 on synchronicity of fluorescence transients between different CA3 areas over all experiments. **(A)** The bar graph indicates that DMSO did not affect the level of synchronization between different CA3 areas (control: 0.74 ± 0.24; DMSO: 0.64 ± 0.28; *n* = 5, ns, unpaired *t*-test). **(B)** The bar graph shows that A484954 enhanced significantly the synchronized activity of different areas of the CA3 region (control: 1.57 ± 0.28; A484954: 0.44 ± 0.18; *n* = 8, ***P < 0.01, unpaired *t*-test).

The average *Z* scores of all control–DMSO and control–A484954 experiments were obtained as outlined previously. The data confirmed that there was no significant difference (*n* = 5, ns, unpaired *t*-test) between control (0.64 ± 0.28) and DMSO application (0.74 ± 0.24, Figure 5A). However, the values of the control and A484954 application were significantly different (*n* = 8, *P* < 0.01, unpaired *t*-test, Figure 5B). The experiments confirmed that under control conditions DMSO application did not affect the neuronal activity pattern and did not alter the synchronization level of fluorescence signals. However, application of the eEF2K inhibitor altered significantly the level of synchronization within different CA3 regions.

The induction of the synchronized neuronal activity by A484954 was also detected using LFP recordings, as outlined in the Supplementary Figures S3A–E. Placing the recording electrode within the CA3 area revealed the coappearance of neuronal activity indicated by the fluorescence transients and LFP oscillations (Supplementary Figure S3B). The fluorescence signals were in a time range of 500 ms; however, the LFPs covered approximately 50 ms with an initial peak followed by smaller activities. The field recording indicated that a burst of activity took place in the CA3 area (Supplementary Figures 3C–E).

**Figure 6:**
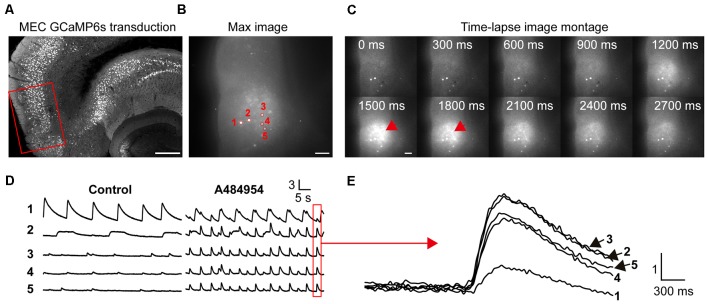
A484954 evoked an increase of cross-correlation in the medial entorhinal cortex (MEC). **(A)** A section of an entorhinal–hippocampal slice that was transduced with GCaMP6s in the MEC is shown. The red rectangle indicates a representative position of the time-lapse fluorescence acquisitions. Horizontal scale bar = 300 μm. **(B)** A representative fluorescence image of the MEC region in a 300-μm-thick acute slice and five ROIs have been presented. Horizontal scale bar = 100 μm. **(C)** A sequence of images of a time-lapse acquisition is shown in a montage. Red arrows indicate increased fluorescence after A484954 application. Scale bar = 100 μm. **(D)** The fluorescence traces for five representative ROIs shown in **(B)** are depicted. Under control condition, only one area of the MEC oscillated, whereas the other four areas showed random activities. After application of A484954, the activity within different ROIs enhanced in a synchronized manner. **(E)** The traces in the red box in **(D)** were overlaid and presented at higher temporal resolution. Vertical scale bar: Δ*F*/*F*_0_.

In the next set of experiments, we studied the effects of A484954 on neurons in the entorhinal cortex (Figure 6A). We focused mainly on neurons in layer II/III (Neves et al., 2008) as depicted in Figure 6B. In this representative experiment, five neurons were analyzed for the purpose of presentation (Figure 6C). The basal activity of the neurons was inconsistent with a regular and strong activity of neuron 1 and small and irregular activities of the other neurons. Their activity became more regular and increased after application of A484954 (Figure 6D). In many cases, the neuronal activity was highly synchronized as indicated in Figure 6E.

**Figure 7:**
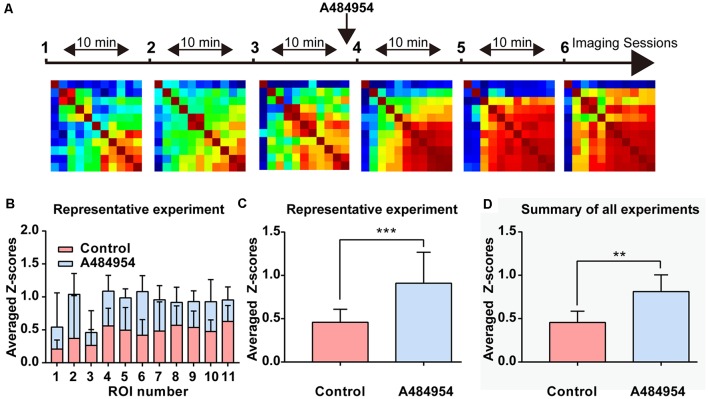
Detailed analysis of the effects of A484954 on MEC neurons. **(A)** The schema indicates the sequence of time-lapse imaging sessions. The first three sessions were under control conditions, and the last three sessions after A484954 application. Every session was a time-lapse acquisition of fluorescence for at least 1.5 min at 33 Hz. The heat maps represent the PCs of all ROI pairs with red larger than 0.8 and blue smaller than −0.8. The PCs increased after drug application gradually. **(B)** The averaged *Z* score (normalized PCs) of each particular ROI over is presented for the two conditions. **(C)** The averaged *Z* scores over all neuronal pairs and three sessions are shown for both conditions. The bar graph indicates the significant effect of the drug on the synchronization of paired fluorescence transients of different ROIs (control, 0.46 ± 0.05; A484954, 0.91 ± 0.10, ***P < 0.01, paired *t*-test). **(D)** The averaged *Z* scores of all neuronal pairs and three sessions were averaged with the values from all experiments (*n* = 4). The drug effect was significant (control, 0.46 ± 0.04; A484954, 0.81 ± 0.06, **P < 0.01, unpaired *t*-test, *n* = 4 slices).

The data analysis of fluorescence transients recorded within different ROIs pattern in another representative experiment confirmed the effects of A484954 on the entorhinal cortex (Figure 7). In these experiments, 11 active cells were observed and analyzed. After application of A484954, the neuronal activity increased and became synchronized as well. This is demonstrated with the Pearson coefficient heat map, where strong correlations of activity are indicated in yellow-red color (Figure 7A). In addition, the averaged *Z* scores of all pairs of an individual ROI over three control or A484954 sessions clearly show the strong effect of the A484954 on synchronized activity. Thus, a significant effect of A484954 on the synchronization level between different ROIs in comparison with drug-free conditions was detectable [control: 0.46 ± 0.05; A484954: 0.91 ± 0.10, *P* < 0.01, paired *t*-test, *n* = 3 session (11 cells), Figure 7C]. The experiments were repeated four times and confirmed the modulatory effect of A484954 (control: 0.46 ± 0.04; A484954: 0.81 ± 0.06, *P* < 0.01, paired *t*-test, *n* = 4 slices).

**Figure 8:**
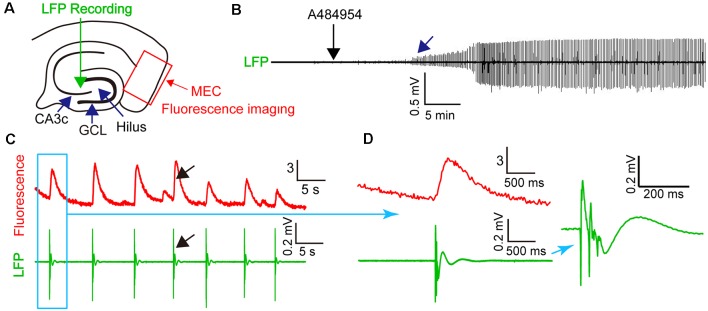
A484954-induced synchronicity of region-specific fluorescence transients in MEC correlated with local field potential (LFP) signals in the CA3 regions. **(A)** Schematic representation of an entorhinal*–*hippocampal slice with its subregions, LFP, and fluorescence recording positions. The overall fluorescence from identified neurons was analyzed. LFP and fluorescence time-lapse recording took place at the same time and slices. **(B)** A representative recording of LFP before and after drug application (black arrow) is shown. The onset of oscillation is indicated by a blue arrow. **(C)** Representative fluorescence (Δ*F/F*_0_) and LFP traces were depicted at the same time scale. A black arrow exemplifies temporally localized appearance of neuronal activity in both fluorescence and LFP channels. **(D)** Transients of fluorescence and LFP of the synchronized event in **(C)** are depicted for different temporal resolutions.

To learn if the synchronized activity of the neurons in the entorhinal cortex is detectable in the CA3 region of the hippocampus, we combined fluorescence imaging in the entorhinal cortex with field potential recordings in the CA3 region (Figure 8A). Application of A484954 induced regular field potentials in the CA3 region (Figure 8B) that were highly synchronized with the overall activity of the entorhinal cortex (fluorescence signal of all active neurons, Figure 8C). The synchronized appearance of fluorescence signal and LFP is also evident at a shorter time interval (Figure 8D). Thus, the activity of entorhinal cortex neurons induced by A484954 might have interfered with the activity of CA3 neurons through their afferents in str. lacunosum moleculare.

### Entorhinal Cortex Afferents to CA3 *via* the Str. Lacunosum Moleculare Remain Intact in Acute Hippocampal–Entorhinal Cortex Slices

**Figure 9:**
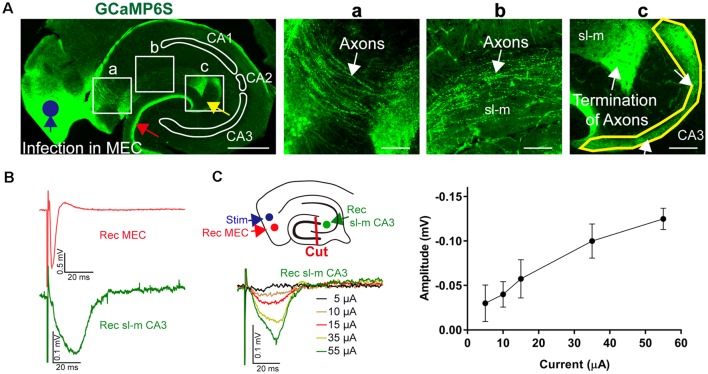
Anatomical and functional evidence of MEC to CA3 connectivity through stratum lacunosum moleculare (sl-m). **(A)** A fluorescence image of a 30-μm section of a representative acute-hippocampal slice depicts the MEC and the hippocampal formation. The MEC was infected with AAV9-hSyn-GCaMP6s (blue circle). MEC axons terminated in the medial pathway of the DG (red arrow) and within the sl-m CA3 region (yellow arrow). Horizontal scale bar: 500 μm. **(a)** Axons (white arrow) are depicted innervating the hippocampus and reaching the sl-m layer. **(b)** The enhanced fluorescence images indicate MEC axons in sl-m (white arrow). **(c)** The termination of MEC axons in the CA3 region (white arrow and yellow outline) is shown. Horizontal scale bar = 100 μm. **(B)** Functional analysis of connectivity was utilized by stimulation at the MEC (layer II/III, blue circle) using a metal electrode and recording in MEC and sl-m of CA3 (green circle) in slices with dissected DG (red line, cut), as shown in the schemata. Representative LFPs are depicted for MEC (red) and sl-m CA3 (green). **(C)** Increase of the stimulation strength resulted in a gradual increase of sl-m CA3 LFPs. The line graph summarizes the stimulation intensity–dependent increase of LFP amplitude (*n* = 4).

To prove that projections from the entorhinal cortex to the CA3 region remain intact in acute *in vitro* slices, we conducted tracing experiments using the *in vivo* overexpression of an anterograde (AV9-hSyn-GCaMP6s) label followed by subsequent standard slice preparations. Overexpression of this tracer in the entorhinal cortex allowed to identify afferents reaching out from the entorhinal cortex along the str. lacunosum moleculare to the CA3c region (Figures 9A,a–c). Most of the afferents terminated in close proximity of the CA2 entrance; however, some afferents formed a thin crescent-shaped layer (Figure 9Ac). This thin crescent-shaped layer reached out from the entrance of the CA2 over the CA3c region to the inner wing of the dentate gyrus (DG; van Groen et al., 2003). The entorhinal cortex afferents also terminated in the medial perforant path of the DG, indicating that the MEC had been overexpressing the anterograde tracers. Both projections to the CA1–CA3 and DG were labeled, because the overexpression of the tracers was not entorhinal cortex layer specific. In addition, the anterograde labeling was useful to confirm the slicing procedure and to recognize the termination area of the MEC axons (Figure 9A). To indicate the functional connectivity of MEC–CA3 over the str. lacunosum moleculare, LFPs were recorded in layer II/III MEC and CA3 in slices with lesioned DG, as outlined in Figure 9B. The input/output characteristic of the MEC-evoked CA3 signal further indicated functional connectivity between these two regions (Figure 9C).

### Functional Confirmation of Entorhinal Cortex Mediated Oscillations in the Hippocampus

The origin or direction of the synchronized neuronal activity is difficult to address because of the limited frame rate of the fluorescence acquisition. Thus, the interconnected pathways from the entorhinal cortex up to the CA1 were subsequently isolated, and the resulting neuronal activity monitored.

**Figure 10:**
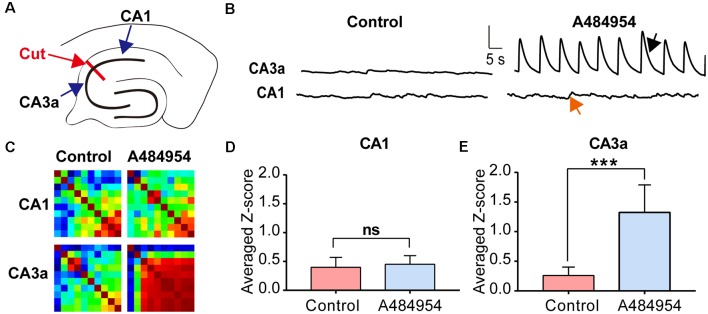
Investigation of the origin of synchronized fluorescence transients in the hippocampal–entorhinal slice after treatment with A484954 by uncoupling CA1 and CA3 region. **(A)** The sketch shows the uncoupling of CA1 and CA3 regions by a cut through the str. oriens, str. pyr, and str. radiatum (red line and arrow). The fluorescence changes were analyzed on both sites of the cut in the CA3 and CA1 regions. **(B)** Representative fluorescence transients are depicted for the indicated regions. Vertical scale bar: Δ*F*/*F*_0_ = 5, horizontal scale bar: 5 s. After uncoupling of CA1 and CA3, a synchronized activity of CA1 neurons after drug application was absent in the CA1 (red arrow) area but not in the CA3 region (black arrow). **(C)** The corresponding heat maps for before and after drug application of one representative experiment represent the same phenomenon as in **(B)**. Whereas under control condition without uncoupling of CA1 and CA3 a synchronization after drug application was detectable, a synchronization was not observable after lesion of the CA1 from CA3 area. However, the CA3 region was still synchronized by the A484954 compound as indicated by the red-colored heat map. **(D)** The bar graph summarizes the result for the CA1 area from different experiments (control: 0.40 ± 0.17; A484954: 0.45 ± 0.15; ns, unpaired *t*-test, *n* = 8). **(E)** The bar graph summarizes the result for the CA3a area (control: 0.30 ± 0.14; A484954: 1.32 ± 0.47; ***P < 0.01, unpaired *t*-test, *n* = 8).

First, we tested if the input from the entorhinal cortex to the CA1 area (Neves et al., 2008) could have triggered the neuronal activity. To this end, the CA1 area was disconnected mechanically from the CA3 area (Figure 10A). Whereas application of A484954 induced synchronized activity in the CA3, a random activity in the CA1 area remained and did not change (Figure 10B). For the CA3 area, an increased coupling of neuronal activity was also evident in the Pearson coefficient heat map (Figure 10C). The summary of eight experiments clearly showed that the neuronal coupling was not enhanced after drug application in the CA1 area (ns, unpaired *t*-test, *n* = 8 slices, Figure 10D). However, the *Z* scores remained significantly elevated in the CA3 area in response to A484954 application (*P* < 0.01, unpaired *t*-test, *n* = 8 slices, Figure 10E). Thus, it becomes evident that the oscillation is not based on the activity of afferents in the str. lacunosum moleculare of the CA1 area, but rather has its origin within the CA3 area.

**Figure 11:**
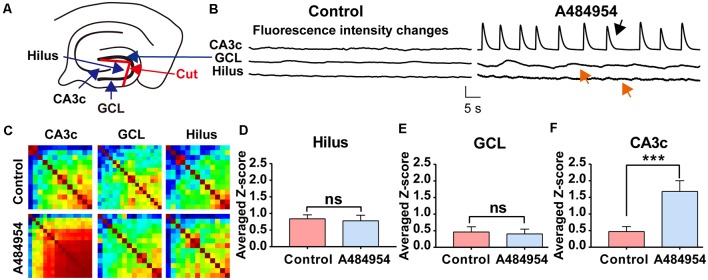
Isolation of the hilus in the DG did not prevent synchronized fluorescence transients in the CA3 region after A484954 application. **(A)** Uncoupling of CA3 and hilus was achieved by a cut between the two wings of the granular cell layer (GCL) as indicated in the sketch (red line and red arrow). **(B)** The drug-induced synchronized activity was observable in the CA3 area (black arrow) but not in the hilus region (red arrow). Vertical scale bar: Δ*F/F*_0_ = 4, horizontal scale bar: 5 s. **(C)** The heat maps for before and after drug application illustrate the different synchronized activity of the analyzed regions. **(D)** *Z*-scores in the Hilus for all corresponding experiments (control: 0.84 ± 0.12; drug: 0.78 ± 0.17; ns, unpaired-*t*-test). **(E)** The bar graph summarizes the *Z* scores in GCL for all corresponding experiments (control: 0.40 ± 0.16; drug: 0.39 ± 0.10; ns, unpaired *t*-test). **(F)** The averaged *Z* scores over all experiments between sessions of control and drug application in the CA3 region differ significantly (control: 0.47 ± 0.15; drug: 1.67 ± 0.32; ***P < 0.01, unpaired-t-test; n = 8).

To further clarify the origin of the A484954-induced activity, the connection between the hilus of the DG and CA3 area was disconnected mechanically (Figure 11A). The rationale behind this experiment was that the entorhinal cortex has a strong innervation in the DG. Thus, the DG itself could be the origin of synchronized activity. However, a significant increase in neuronal activity after A484954 application was never observed. Even more, the disconnection of the DG hilus from the CA3 region did not prevent the synchronized activity within the CA3 (Figure 11B). The heat maps of the Pearson coefficients also indicated that a neuronal coupling took place in the CA3 area after drug application, but not in the hilus (Figure 11C). Repetition of the experiment several times clearly showed that drug-mediated synchronization of CA3 neurons remained significantly increased even after the uncoupling of the hilus from the CA3 region (Figure 11F). However, the neuronal activity in the hilus and GCL was not altered after drug application (Figures 11D,E).

**Figure 12:**
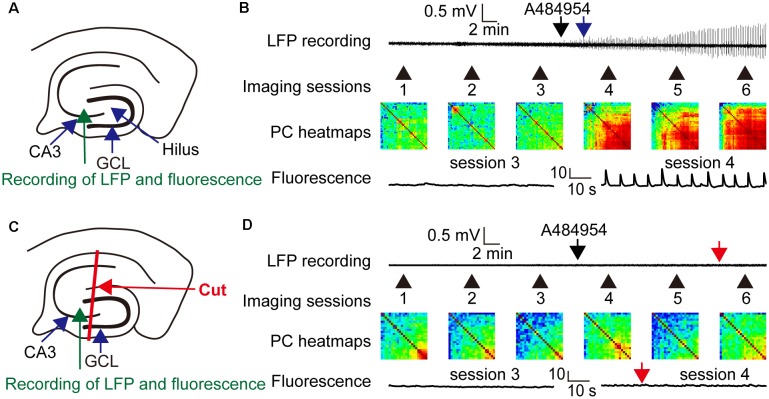
Drug effects on LFPs and fluorescence signals in coupled and uncoupled entorhinal–hippocampal acute slices. **(A)** Schematic representation of LFP and fluorescence recording sites in the CA3 area (green arrow). **(B)** A representative LFP trace has been shown indicating the time point of drug application (black arrow) and the onset of oscillation (blue arrow). In the same experiment, the fluorescence changes were acquired six times. Every session took place as indicated with the numbered black arrows. The intersession interval was 10 min. The heat map indicates the gradual synchronization of the fluorescence transients between different pairs of ROIs. In the heat map of session 6, most pairs correlated in their activity with a high PC (>0.8, yellow–red). Representative fluorescence traces have been shown for sessions 3 and 4 below the heat map (Δ*F/F*_0_: vertical scale bar). **(C)** Schematic representation of LFP (green arrow) and fluorescence recording (blue arrow) sites in the CA3 area. The slice was lesioned along str. oriens-sl-m of the CA1 region, through the hilus up to the outwing of the DG region. This lesion uncouples the CA3 region from entorhinal inputs. **(D)** The outline of the panel follows the outline presented in **(B)**. The red arrows indicate the absence of any LFP oscillation or fluorescence signals in the entorhinal-hippocampal uncoupled slice. In addition, the PC heat map did not indicate an enhancement of cross-correlation coefficients. Thus, the experiments demonstrated a dependency of drug induced oscillation or synchronization of neurons by the specific entorhinal fiber bundle that terminates in the region of CA3–CA2 in the sl-m.

The entorhinal afferents toward the CA3 area were considered because the DG and CA1 area were not involved in the drug-mediated enhancement of neuronal activity. To this end, drug-mediated effects were compared between intact slices (Figure 12A) and slices with mechanically separated CA3 area that did not process inputs from the CA1 str. lacunosum moleculare and hilus as outlined in Figure 12C. In addition, LFPs and fluorescence changes were recorded simultaneously within the CA3c area. Application of A484954 induced a clear oscillation of LFPs and a strong coupling of neuronal activity (Figure 12B) in the intact slice. This was also evident in the heat maps of Pearson coefficients in sessions 4–6 (Figure 12B). However, application of A484954 to slices with a dissected CA3 area (Figure 12C) prevented the induction of LFP oscillations and the enhancement of rhythmical fluorescence oscillations (Figure 12D). Thus, the afferents from the entorhinal cortex to the CA3 area are required for A484954-mediated enhancement of neuronal activity in the hippocampal slice.

**Figure 13:**
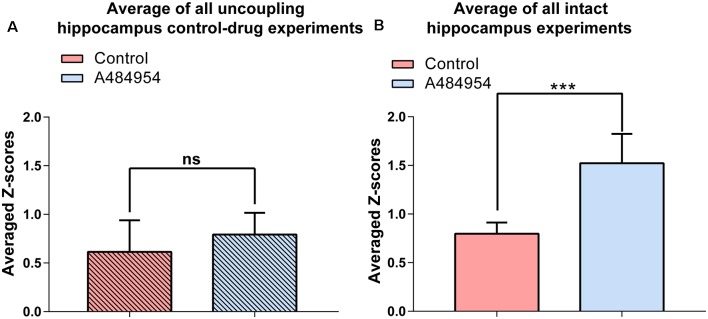
A484954 did not induce synchronized fluorescence transients within different areas of the CA3 region in hippocampal slices upon uncoupling the CA3 region from the entorhinal inputs. **(A)** The bar graph summarizes that A484954 did not activate synchronized fluorescence transients in the CA3 region of slices after uncoupling of the CA3 region from entorhinal inputs (control: 0.61 ± 0.14; A484954: 0.79 ± 0.10; ns, unpaired *t*-test, *n* = 5 slices). **(B)** The bar graph shows that A484954 enhanced significant synchronization between different areas of the CA3 region in the intact hippocampus (control: 0.80 ± 0.05; A484954: 1.52 ± 0.12; *n* = 6, ***P < 0.01, unpaired *t*-test, *n* = 6 slices).

Statistical analysis of the A484954 effects in hippocampal slices with intact entorhinal cortex–hippocampal circuits and slices with dissected CA3 region further proved the role of the entorhinal cortex in this drug-mediated enhancement by A484954 (Figure 13). Whereas the *Z* score of the Pearson coefficient remained within the same level after drug application in the uncoupled slices (ns, unpaired *t*-test, *n* = 5 slices), the *Z* scores over all experiments increased significantly in the intact hippocampal slices (control: 0.80 ± 0.05; A484954: 1.52 ± 0.12; *n* = 6, *P* < 0.01, unpaired *t*-test, *n* = 6 slices).

## Discussion

It has been shown that A484954, a compound that inhibits eEF2K activity, induces slow-onset potentiation of hippocampal synaptic transmission (Weng et al., 2016). Here, we presented additional data showing that A484954 evokes a synchronized activity of neurons within the hippocampus and entorhinal cortex in acute hippocampal–entorhinal slices. The observed activation pattern depends on intact afferents originating from the entorhinal cortex to the CA3 area. The experiments also point to the fact that defining region-specific drug mechanisms are indeed very complex because of the subregion-dependent influences.

### Transfection of Hippocampal Neurons With GCaMP6s

All the experiments were performed using hippocampal–entorhinal cortex slices (Leutgeb et al., 2005). The hippocampal slices were prepared at an angle that preserves the reciprocal connections between the entorhinal cortex and the hippocampus *in vitro* (Rafiq et al., 1993; Xiong et al., 2017). In this way, afferents from the entorhinal cortex to the CA3 neurons (Neves et al., 2008) remained functional in acute slices. We also confirmed the presence of entorhinal cortex–hippocampal afferents in acute slices utilizing tracing experiments (Tervo et al., 2016; Yamamoto et al., 2018) and dual recordings from entorhinal cortex and CA3 area. These data are in line with another publication demonstrating the presence of intact entorhinal cortex to hippocampus afferents in the str. lacunosum moleculare by a lipophilic fluorescent tracer in fixed acute hippocampal slices (Xiong et al., 2017).

Chemical and genetically encoded calcium indicators are utilized for the analysis of neuronal activity (Grienberger and Konnerth, 2012). Here, we exploited the genetically encoded calcium indicator GCaMP6s to detect intracellular calcium changes in response to single or bursts of action potentials (Chen et al., 2013). The expression of GCaMP6s was aimed to take place mainly in hippocampal neurons utilizing the adeno-associated virus serotype-specific infection pattern (Watakabe et al., 2015) and a CaMKIIα promoter-driven expression (Wang et al., 2013).

Spontaneous neuronal activity has been observed in acute hippocampal slices (Hájos et al., 2013; Butler and Paulsen, 2015; Maier and Kempter, 2017) that could be attenuated by cholinergic or serotonergic stimulations (Kubota et al., 2003). However, irregular epileptiform discharges might on top be generated that originate from the entorhinal cortex and/or DG similar to that observed in acute brain slices from temporal lobe epilepsy models (Weissinger et al., 2017). We performed at least three imaging sessions under drug-free conditions to ensure a low level and stability of neuronal activity. Other *in vivo* and *in vitro* studies also observed that excitatory neurons in the CA1 or CA3 area possess little activity and tend to activate independently of each other (Takano et al., 2012; Modi et al., 2014; Hainmueller and Bartos, 2018).

### A484954 Enhanced Hippocampal Neuronal Activity, Which Is Dependent on Afferents From the Entorhinal Cortex to the CA3 Region

A484954 is an eEF2K inhibitor (Chen et al., 2011) that reduces the degree of eEF2 phosphorylation leading to an enhancement of protein synthesis (McCamphill et al., 2015). However, as we also have described before, some of the A484954 effects might be mediated by other related or even nonspecific effects (Weng et al., 2016). Administration of this compound enhanced hippocampal synaptic transmission and increased the neuronal activity of cultured hippocampal neurons (Weng et al., 2016). In acute hippocampal slices, an enhancement of neuronal activity and their synchronization occurred within several minutes after adding A484954. Interestingly, the enhancement of neuronal activity by A484954 took place in the CA3–CA1 regions, but not within the DG. Different regions of the hippocampus were mechanically uncoupled to distinguish the origin of the synchronized activity (Barbarosie and Avoli, 1997; Barbarosie et al., 2000). The lesions took place at the borders of CA3/CA2 to CA1, or hilus to CA3, or GCL of DG to hilus. In addition, the time-lapse fluorescence imaging was performed on both sides of the adjacent, but separated areas. As we have shown, the neuronal activity was not enhanced in the separated areas in the direction of the trisynaptic circuit (Neves et al., 2008) of the hippocampal formation (CA3–CA1). Furthermore, the isolation of the hilus from the CA3 or granular cells from the hilus did not alter the activity pattern within different ROIs in the adjacent CA3 area.

The hippocampal formation is tightly interconnected with the entorhinal cortex (Basu and Siegelbaum, 2015). Therefore, the neuronal rhythm generated in the hippocampus might contribute to the neuronal activity in the entorhinal cortex or vice versa (Barbarosie and Avoli, 1997; Barbarosie et al., 2000; Basu and Siegelbaum, 2015; Yamamoto and Tonegawa, 2017). Some studies have highlighted a strong entorhinal cortex-driven regulation of neuronal activity in the hippocampal formation through the DG (Barbarosie and Avoli, 1997; Barbarosie et al., 2000) or the activation of CA1 neurons through the entorhinal cortex afferents in the str. lacunosum moleculare (Basu et al., 2016; Yamamoto and Tonegawa, 2017). However, in our experiments, where the CA3 area was lesioned from the CA1 and DG regions, a significant attenuation of the A484954 effect on neuronal activity was observable. Whereas the neuronal activity in the entorhinal cortex remained enhanced and synchronized, the neurons in the CA3 as well as CA1 area (Figure 10A) did not exhibit any changes after A484954 application. Thus, the experiments suggested that A484954-mediated synchronization of neural network activity is facilitated by entorhinal afferents terminating at the distal dendrites of the CA3 region in the str. lacunosum moleculare. In conjunction with the other lesion experiments, we concluded that the action of the entorhinal afferents is mediated through some effects on cellular components within the distal part of the CA3 area.

It has been shown that the density of GABA receptors in the termination area of entorhinal afferents in the CA3 area expresses an elevated level of GABA_B_ receptors (Kulik et al., 2003). Especially the termination area of the entorhinal cortex afferents in str. lacunosum moleculare of the CA3 has been found to contain a high GABA_B2_ expression with presynaptic but also postsynaptic locations (Kulik et al., 2003). In addition, long-range projections of GABAergic neurons originating in the entorhinal cortex have been described that regulate oscillatory activity in the hippocampal target areas including CA3 region (Melzer et al., 2012; Basu et al., 2016). Whether A484954 interacts with the GABA_B_ receptors in the hippocampus or is altering the activity of GABAergic neurons in the entorhinal cortex remains to be studied. In addition, other studies have shown that inhibition of eEF2K modulates GABA-mediated synaptic transmission and is affecting the balance of inhibitory and excitatory synaptic transmission (Weng et al., 2016; Heise et al., 2017). However, in our previous study, we described that the A484954-mediated enhancement of hippocampal synaptic transmission relies on the modulation of presynaptic mechanisms (Weng et al., 2016). Therefore, we speculate that A484954 modulates neuronal activity by increasing excitatory synaptic transmission. To distinguish the mechanism underlying A484954 effects on neuronal network properties is challenging because inhibition of the GABAergic system by a specific antagonist will increase the likelihood of burst discharges by itself. At any rate, as we have shown, the up-regulation of neuronal activity and its synchronization rely on the activity of entorhinal cortex inputs (Basu and Siegelbaum, 2015). However, rhythmic neuronal activity (theta, gamma) can be also initiated and maintained by neuronal assemblies in the hippocampal formation that create rhythm generators. Modulation of the inhibitory and excitatory balance by activation of acetyl cholinergic, kainic, *N*-methyl-D-aspartate, or metabotropic receptors can further enhance the activity of such rhythm generators in the CA3 and CA1 area without the contribution of the entorhinal cortex (for review, see Butler and Paulsen, 2015).

Overall, this study was focused on structural relevance of an MEC to CA3 connectivity, bypassing the DG. However, in the future, additional research is required to decipher the modulations that might take place within the MEC and sl.-m. CA3 in response to the A484954 application. This might also imply the use of mesoscopic imaging systems (Behnisch et al., 2004) and more cell-specific analyses of synchronized activity using different statistical methods (Bastos and Schoffelen, 2015) or imaging analysis packages (Giovannucci et al., 2019; Pachitariu et al., 2018).

### Modulation of Neuronal Oscillations by eEF2K Inhibition May Contribute to the Study of Antidepressant Treatments

Ketamine and ketamine derivatives can cause a rapid antidepressant effect (Yao et al., 2018; Krystal et al., 2019; Zanos et al., 2019). Studies have linked the rapid antidepressant effect with the modulation of eEF2K, causing an increase in protein translation, like brain-derived neurotrophic factor (BDNF; Monteggia et al., 2013). In addition, ketamine alters neuronal network activity within the brain (Yang et al., 2018; Zanos et al., 2019) that might contribute to its antidepressant effects. This idea is based on the hypothesis that synchronized oscillations within different brain areas contribute considerably to information processing (Engel et al., 2001; Varela et al., 2001; Fernández-Ruiz et al., 2017) and certain forms of mood disorders (D’ostilio and Garraux, 2016). Further *in vivo* experiments are needed to prove that direct inhibition of eEF2K or administration of A484954 in the hippocampal brain area *in vivo* can also exert a rapid antidepressant effect by modulation of the neuronal activity pattern.

## Conclusion

In summary, the inhibition of eEF2K in the hippocampal–entorhinal cortex system alters the intrinsic activity of interconnected neuronal microcircuits that is dominated by the MEC–CA3 inputs. In addition, the experiments make aware of the complexity to uniquely define region-specific drug mechanisms due to the subregion-dependent influences of drug effects. The effects of drugs such as A484954 on the oscillation pattern of multiple subregion-specific neuronal microcircuits might worthily represent a potential drug research strategy to combat mood disorders.

## Data Availability Statement

The datasets generated for this study are available on request to the corresponding author.

## Ethics Statement

The animal study was reviewed and approved by Institutional Animal Care and Use Committee of Fudan University, Shanghai Medical College (IACUC Animal Project Number: 31320103906).

## Author Contributions

ZL, CP, YZ, YC, and TB apprehended the project and designed the experiments. ZL and CP carried out the experiments and analyzed the data with the help of YZ and YC. ZL, CP, YC, and TB wrote the manuscript.

## Conflict of Interest

The authors declare that the research was conducted in the absence of any commercial or financial relationships that could be construed as a potential conflict of interest.
