# Supplementary material for: Direct Medial Entorhinal Cortex Input to Hippocampal CA3 Is Crucial for eEF2K Inhibitor-Induced Neuronal Oscillations in the Mouse Hippocampus
Source: Front Cell Neurosci. 2020 Mar 6;14:24. doi: 10.3389/fncel.2020.00024 (PMC7069380; doi:10.3389/fncel.2020.00024)
Supplement: Supplementary file 1 [file Table_1.docx]

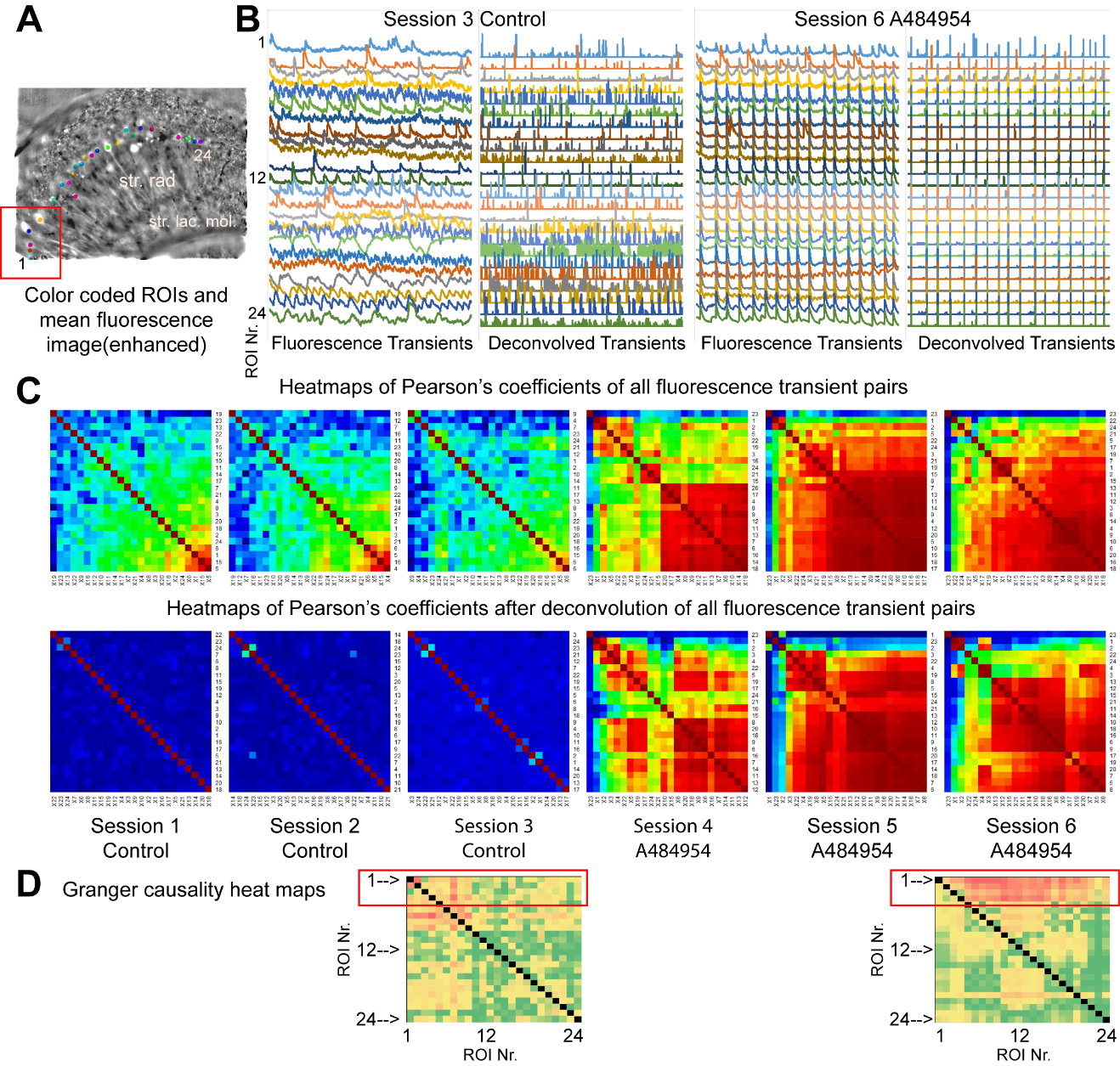


**Supplementary Figure S1. Reanalysis of the figure 2 data set using Suite2p, deconvolution, and Granger-causality analysis.**

1. The image represents the enhanced mean image of the Suite2p output (Python, Suite2p, https://doi.org/10.1523/JNEUROSCI.3339-17.2018). ROIs (24) were placed manually along the CA3/CA2-CA1 str. pyramidale to cover fluorescent cell bodies, starting with the first ROI at the left lower corner (red square, first four ROIs within the end of CA3 region). The positions of the ROIs were kept constant over all sessions.
2. Min-Max scaled fluorescence (F-Fneuropil*0.7) and corresponding deconvolved transients (according of Suite2p manual) before and after A484954 application are depicted. Image acquisition took place at 33 Hz and 3000 single frames (640x540 pixels) were captured.
3. The Pearson coefficient for all possible pairs of ROIs are presented in heat maps (blue: -1 to red: 1, clustered). The first row of heat map shows the results using values of the fluorescence transients, and the second row the heat maps for the deconvolved transients. The deconvolution estimates the degree of neuronal activity and removes drifts/trends of fluorescence transients. Application of A484954 (Session 4-6) induced an increase of the number of ROI pairs with high Pearson’s coefficient values in each of the data analysis.
4. To estimate the causal interaction of ROI pairs a Granger analysis of deconvolved transients (Spatio-temporal Granger Causality; Matlab, V2.0, //doi.org/10.3389/fnhum.2017.00513) has been performed. The causality values (direction y to x: e.g. ROI 1🡪ROI 2-24) for session three and six were depicted in a heat map (green (0) <yellow (0.4) <red (1)). One interpretation of the data is that the distribution of red cells in the heat map indicated that the activity within the first four ROIs (CA3/CA2) preceded/ granger-caused activity of the remaining ROIs (CA1).


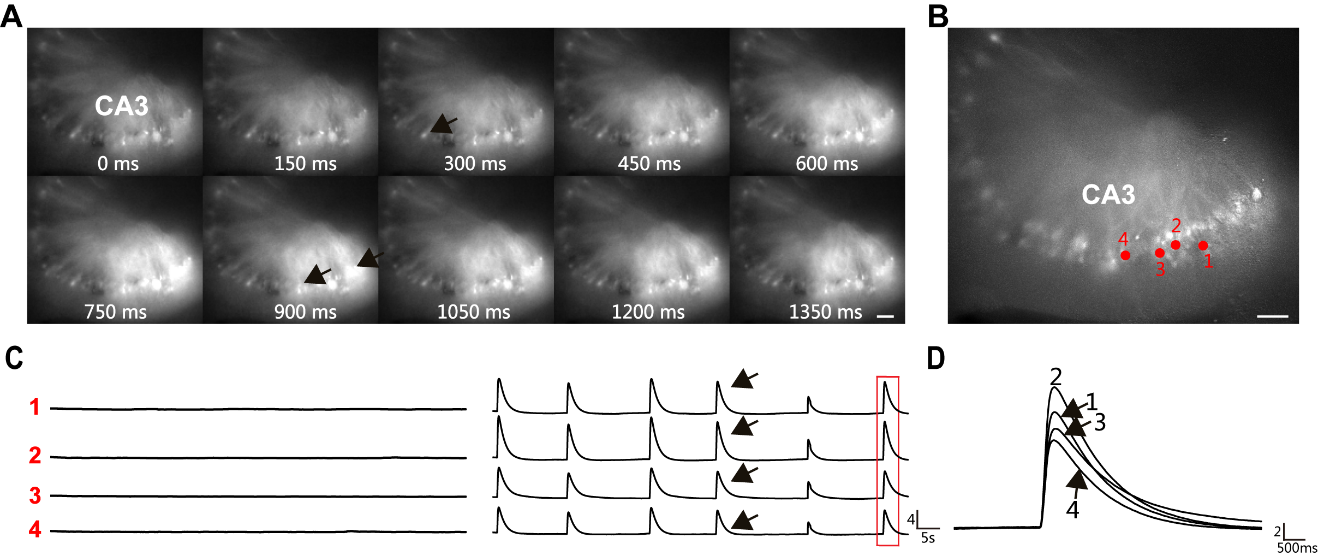


Supplementary Figure S2. A484954 induces synchronized neuronal activity in the hippocampal CA3 region.

1. An image montage with a time interval of 150 ms of a representative time-lapse acquisition is shown. Scale bar=100 µm. The drug A484954 (10 µM) was applied before the recording session. The white areas (black arrows) indicate regions of synchronized activity (e.g. 300 ms and 900 ms).
2. For presentation of fluorescence intensity transients, four ROIs were chosen (red filled circles). Horizontal scale bar=100 µm.
3. The fluorescence intensity transients for the four ROIs under control and drug conditions are presented. Under control conditions, only little spontaneous activity was detectable. However, after application the larger part of the CA3 developed synchronized neuronal activity (black arrows). The transients in the red rectangle are shown in D. Vertical scale bar: △F/F_0_ = 4, Horizontal scale bar = 5 s.
4. The transients indicate the temporal proximity of the events. Vertical scale bar: △F/F_0_ = 2, Horizontal scale bar = 500 ms


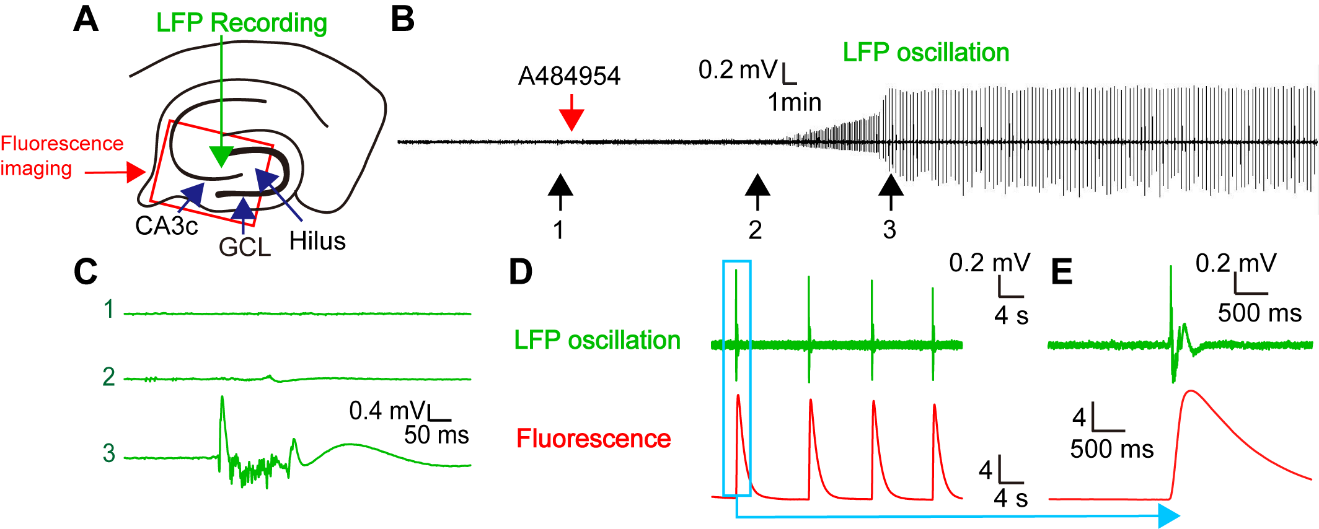


Supplementary Figure S3. Synchronized fluorescence signals after treatment with A484954 correlated with local field potential (LFP) in the CA3 pyramidal layer.

1. The schematic outlines the relative position of the local field potential (LFP) recording site (green arrow) and the fluorescence calcium imaging area (CA3c).
2. Traces of a representative LFP recording has been shown. The application time of A484954 is indicated with a red arrow. The oscillation started several minutes after drug application in the CA3c area. Arrows with green numbers indicate before drug application (1), represents the beginning of LFP oscillation, (2) and shows the LFP oscillation at its maximum (3). The corresponding traces with a smaller time scale are shown in C). Vertical scale bar = 0.2 mV; Horizontal scale bar = 1 min.
3. Representative traces with a smaller time scale of time point 1 to 3 are depicted. The burst of an oscillation is about 100 to 200 ms.
4. Combined fluorescence imaging and LFP recording indicated the temporal co-localization of the events at time point 3. The bursts of the oscillation take place every 10-15 s.
5. LFP and fluorescence traces within the blue box in D at higher temporal resolution are presented.
